# Timing of social feedback shapes observational learning in strategic interaction

**DOI:** 10.1038/s41598-021-01466-1

**Published:** 2021-11-09

**Authors:** Joshua Zonca, Alexander Vostroknutov, Giorgio Coricelli, Luca Polonio

**Affiliations:** 1 Cognitive Architecture for Collaborative Technologies (CONTACT) Unit, Italian Institute of Technology, Genoa, Italy; 2 Department of Economics (MPE), Maastricht University, Maastricht, The Netherlands; 3 Department of Economics, University of Southern California, Los Angeles, USA; 4 Molecular Mind Laboratory (MoMiLab) Unit, IMT School for Advanced Studies Lucca, Lucca, Italy

**Keywords:** Human behaviour, Decision, Social behaviour, Learning and memory

## Abstract

Many types of social interaction require the ability to anticipate others' behavior, which is commonly referred to as strategic sophistication. In this context, observational learning can represent a decisive tool for behavioral adaptation. However, little is known on whether and when individuals learn from observation in interactive settings. In the current study, 321 participants played one-shot interactive games and, at a given time along the experiment, they could observe the choices of an overtly efficient player. This social feedback could be provided before or after the participant’s choice in each game. Results reveal that players with a sufficient level of strategic skills increased their level of sophistication only when the social feedback was provided after their choices, whereas they relied on blind imitation when they received feedback before their decision. Conversely, less sophisticated players did not increase their level of sophistication, regardless of the type of social feedback. Our findings disclose the interplay between endogenous and exogenous factors modulating observational learning in strategic interaction.

## Introduction

Social interaction is a core aspect of human behavior that sustains the multifaceted social systems underlying our society. In many social environments, the outcomes of our decisions are influenced by the actions of other actors, and our behavioral success depends on the ability to foresee peers’ behavior and best respond to it. We refer to this ability as strategic sophistication. Strategic sophistication plays a crucial role in many small and large scale social contexts including job markets, technology races, financial markets and economic growth plans. Nonetheless, extensive evidence has shown that humans rarely implement perfectly rational and strategic behavior in social settings^1^. This bounded (strategic) rationality can lead to negative consequences for individuals and organizations that are not able to correctly anticipate the behaviour of other actors (e.g., citizens, competitors) in their social environment and proactively make strategic decisions. In this regard, one of the most fascinating questions on social interaction is whether and how strategic sophistication can be enhanced to maximize behavioral efficacy in social settings.

One possibility for the development of strategic skills entails the exposure to feedback on the outcomes of ongoing interactions^2^. In case of negative outcomes, we can then update our beliefs on the future actions of our counterpart(s) and adapt our own strategy accordingly^3,4^. Nevertheless, in our everyday experience, social interactive contexts do not often provide straightforward and accurate feedback on the current interaction. For instance, our counterparts may be reluctant to disclose their future actions and goals to prevent us to take advantage of this information. In these uncertain environments, observing the actions and the relative outcomes of other actors interacting in the same (or in a similar) social environment, namely observational learning, may represent an alternative valuable resource for learning strategic skills. Observational learning and social comparisons are fundamental components of human behavior^5^ and evolution^6–8^. Through observational learning, individuals have the opportunity to learn and implement successful behavioral strategies minimizing risks^9,10^, costs^8,11^ and uncertainty^12,13^.

A peculiar feature of observational learning is that the observed behavior might be acquired and learned even *before* performing an action in a given environment. For instance, when unsure about what action to take, we may observe the behavior of someone else who is known to be successful in that context. This observational feedback can be used in two different ways. On the one hand, we can imitate blindly the observed actions without understanding the reasons underlying them^10,14–16^. Alternatively, we might try to understand the rationale behind the observed behavior and use this information to learn new efficient behaviors^8,13,17–19^. When humans have the possibility to select between these two learning strategies, they do so depending on their capabilities, cognitive costs and contextual goals^20^. The former strategy minimizes computational costs^9,21^ and is extremely efficient as long as the actions of the behavioral model are available and replicable. However, imitation prevents generalization in the presence of changes in the environment, since the acquired information may be outdated and no longer efficient^22,23^. The latter strategy is computationally heavier, but entails a deep understanding of the motivations that make the observed strategy efficient and allows its future application in new contexts.

Nonetheless, in social settings we do not always have the possibility to be guided by others’ behavior and we may be urged to make a choice without external guidance. In particular, in some cases we have the opportunity to observe the behavior of successful agents only *after* we have made our choices in that environment. In these contexts, observational learning occurs with the same modalities of asocial learning, relying on reinforcement mechanisms. For instance, we can compare own and others’ behavior and, if the observed behavior is more efficient, adjust our strategy to fit the peculiar characteristics of the successful one. This process can be described by computational models expressing the integration of others’ choices as a social (or action) prediction error, which represents the discrepancy between the expected and the actual choice of the observed actor^10,24^. By preventing the reliance on imitative behavior, these kinds of interactive contexts may exogenously promote the emergence of sophisticated learning mechanisms allowing the transfer of (socially) acquired knowledge to future interactive contexts, even in the presence of environmental changes.

**Figure 1:**
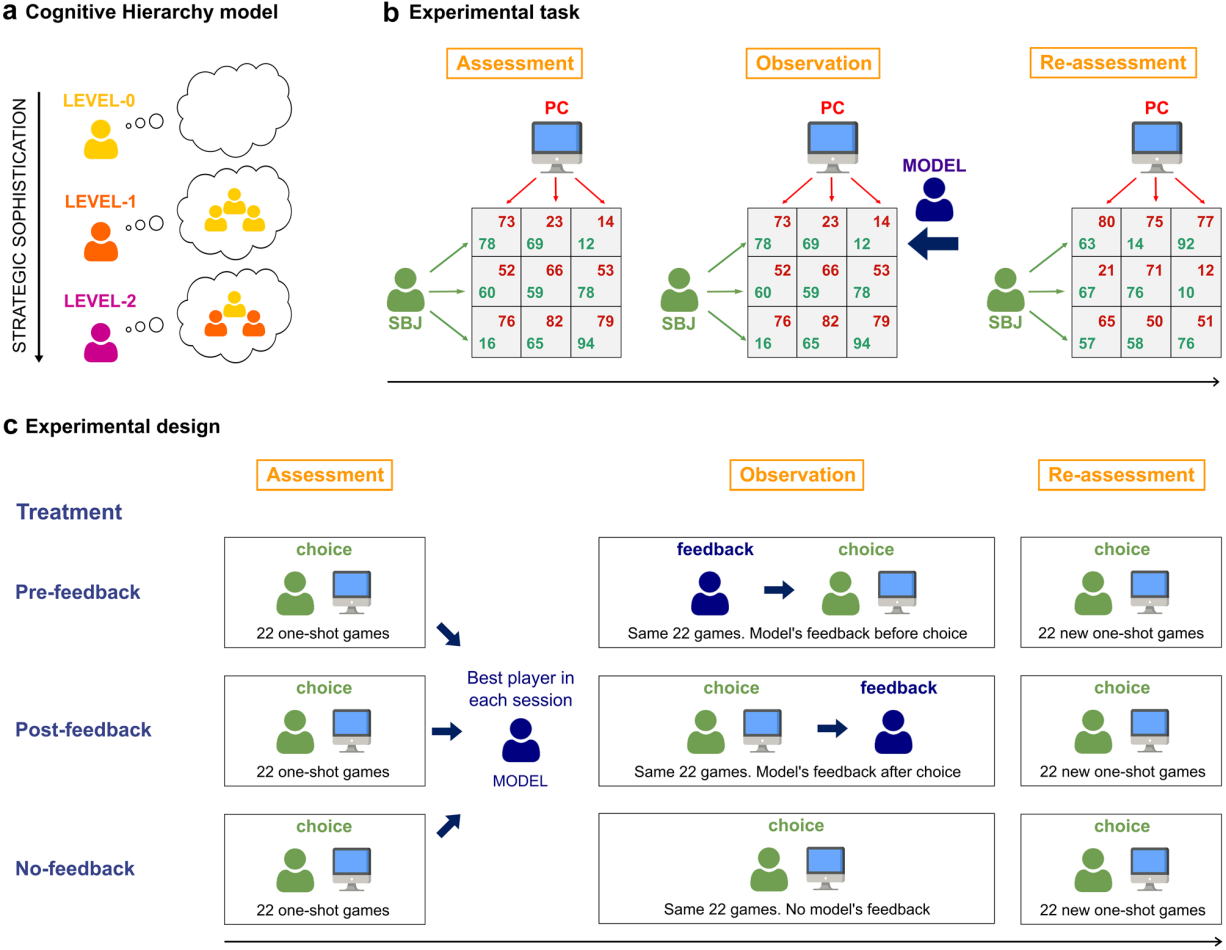
(**a**) Graphical illustration of the Cognitive Hierarchy (CH) model. CH models the strategy space of players through a hierarchical structure characterized by increasing levels of strategic sophistication. The hierarchy starts with players who play randomly and do not form any beliefs about the choices of their counterparts (level-0). The second level in the hierarchy predicts level-1 players, who best respond to the belief that the counterparts are level-0; then CH predicts level-2 players, who best respond to the belief that the population of potential opponents is a mixture between level-0 and level-1, and so on, increasing the number of steps of strategic sophistication. (**b**) Experimental task. Participants were tested in groups. Every participant underwent three consecutive experimental phases (Assessment, Observation and Re-Assessment). In all phases, participants played one-shot 3 × 3 normal form games with a computer algorithm (PC) with fixed but unknown behavior (level-1 strategy). Participants played as row player and had to select one of the three rows of the game matrix, whereas the artificial agent played as column player and chose one of the matrix columns. The combination of the two players’ choices determined the game outcome (green payoff for the participant, red payoff for the artificial agent). Participants did not receive feedback on the game outcomes. In the Observation phase, participants received feedback on the choices of the best player in the Assessment phase (the model). The feedback consisted in a black arrow in correspondence to the row selected by the model. (**c**) Experimental design. Participants in a specific experimental session were assigned to one of three experimental treatments (No-feedback, Pre-feedback, Post-feedback). The Assessment phase was identical for all the experimental treatments: participants played 22 one-shot games with the same artificial agent, without any feedback. Then participants in all treatments were told if they were the best player in the session (or not). In the Observation phase, participants received different types of feedback based on the experimental treatment. In the Pre-feedback treatment, participants could observe the decision taken by the model (the best player in the Assessment phase) in that game as soon as the game matrix appeared, *before* they made their choice. Conversely, participants in the Post-feedback treatment could see the model’s feedback only *after* they made their decision. In the No-feedback treatment, participants did not receive any feedback on the model’s choices. Eventually, participants in all treatments underwent the Re-assessment phase, in which they played 22 new games without receiving the model’s feedback, as in the Assessment phase.

Despite the potential impact of observational learning on behavioral adaptation in social interaction, we lack evidence highlighting whether and in what circumstances humans learn from observation in interactive settings. Our study aims at investigating if individuals can improve their level of strategic sophistication by observing a successful agent and whether the timing of social feedback (i.e., before or after choice) can shape the emergence of sophisticated and generalized learning. To explore these hypotheses, we used game theory as a behavioral model of social interaction. Specifically, we employed one-shot normal form games, which are usually used to model strategic interaction between two players. Normal form games consist in a set of actions and incentives (i.e. payoffs) for each player: each player makes a choice and then the combination of the two players’ choices determines their relative outcomes. We characterized heterogeneity in strategic sophistication by analyzing strategic behavior in the framework of the Cognitive Hierarchy (CH) model^25–27^. This model describes choice behavior in terms of hierarchical levels of strategic thinking. Each player tries to predict the strategic level of the counterpart(s) and then best respond to this belief by employing a more sophisticated strategy (Fig. 1a). The hierarchy starts from players who play randomly (level-0); then assumes level-1 players, who best respond to the believe that all other players are level-0; then it predicts level-2 players, who believe that the population of potential opponents is not more sophisticated than level-1, and so on, increasing the number of steps of strategic thinking. This theoretical framework assumes heterogeneity in individuals’ strategic sophistication, in contrast with Nash equilibrium^28^, which postulates perfect rationality of agents that have consistent beliefs about others’ upcoming actions and make the best choice given their expectations^29^. This heterogeneity can be explained by different factors, which can be grouped in two major categories. First, heterogeneity in strategic thinking is linked to variability in participants’ beliefs about others’ level of strategic thinking. Second, the implementation of complex strategies involving more than one step of strategic thinking requires different cognitive abilities, including mentalizing, fluid intelligence, working memory, cognitive reflection and representation skills, as revealed by extensive experimental evidence [e.g.,^30–33^]. Individuals with less sophisticated cognitive abilities, without proper external training and guidance, may struggle in autonomously generating or understanding sophisticated strategies in social interaction^34,35^.

The experimental design (Fig. 1b,c) consisted in three within-subject consecutive phases (Assessment, Observation, Re-assessment) and three between-subject experimental treatments (No-feedback, Pre-feedback, Post-feedback). Three hundred twenty one (321) participants performed the task in groups. In the Assessment phase, participants in all treatments played 22 games with an artificial agent playing a fixed behavioral strategy, unknown to participants, across all games. Participants were told that this artificial agent would play, in each game, the most common strategy used by real human players in those types of game^35–37^. This aspect of the experimental design is important since, in environments with multiple unknown potential partners, behavioral fitness depends on the ability to best respond to the most common behavior in the population^4,38,39^. The strategy used by the artificial agent is the one of an unsophisticated level-1 player: this agent selects the action with the highest average payoff for itself, assuming the counterpart to play randomly (See “Games and artificial agent behavior” paragraph in the “Methods” section for a detailed description of the games used in the experiment and the artificial agent’s behavior). Another important aspect of the Assessment phase is that no feedback on the game outcomes was provided to participants: therefore, they could not learn from the behaviour of the artificial agent.

Then participants underwent the Observation phase, in which they played again the same 22 games with no outcome feedback. However, in each game, they could receive additional information about the choice taken, in the Assessment phase, by the player who accumulated the higher score in the Assessment phase in the current experimental session (i.e., the *model*). The experimental treatment prescribed what type of feedback participants received in the Observation phase. In the No-feedback treatment, which served as baseline, participants did not receive any feedback from the model. In the Pre-feedback treatment, participants could observe the decision taken by the model in that specific game *before* making their decision. On the contrary, participants in the Post-feedback treatment received the feedback about the model’s decision only *after* they made their decision and could not modify their choice based on the model’s feedback.

Right after the Observation phase, participants underwent the Re-assessment phase, in which they played 22 *new* games without any feedback from the model or on the game outcomes, as in the Assessment phase. We highlight that participants knew about the existence of the three experimental phases from the start of the experiment.

In our analyses, we compare participants’ strategic behavior in Re-assessment and Assessment phase across treatments to investigate the emergence of different forms of social learning depending on the timing of social feedback. Moreover, since the ability to learn to play strategically may be linked to the possession of fundamental skills underlying strategic behavior, we analyze the emergence of observational learning as a function of the participants’ initial level of strategic thinking, as measured through choices in the Assessment phase.

First, we hypothesize that the ability to enhance strategic behavior through observational learning may require an adequate (initial) level of strategic thinking. Players starting form a low level of strategic sophistication in the Assessment phase may not succeed in grasping the fundamental features of the model’s behavior in the Observation phase, independently of the experimental treatment. This might be explained by low cognitive abilities, in line with evidence highlighting a relationship between strategic sophistication and cognitive skills^30–35^. Conversely, for participants with a sufficient initial level of strategic sophistication, we hypothesize that the ability to apply sophisticated (and cognitively costly) observational learning strategies strictly depends on the timing of social feedback and the availability of costless shortcuts such as (blind) imitation. In particular, we predict that receiving the model’s feedback only after making choices (Post-feedback treatment) could boost sophisticated forms of social learning, leading to an increase in participants’ levels of strategic sophistication in the Re-assessment phase. On the contrary, having access to the model’s feedback before choosing (Pre-feedback treatment) could encourage (blind) imitative behavior, preventing generalization of the observed behavior in novel and unguided contexts and, therefore, strategic improvement in the Re-assessment phase.

## Results

### Exploring heterogeneity in strategic sophistication

In line with previous findings in similar games^35–37^, participants rarely played the Nash equilibrium strategy in the Assessment phase. The average proportion of equilibrium choices in our sample is rather low (0.40 ± 0.14) and drops drastically when excluding games in which the Nash equilibrium solution coincides with the level-2 choice (0.18 ± 0.11). These results indicate that the Nash equilibrium model alone is a poor predictor of participants’ strategic behavior in this type of games. Therefore, we aimed at characterizing participants’ heterogeneity following the hierarchical structure of the Cognitive Hierarchy (CH) model (Fig. 1a). We used the level-2 strategy, which represents the optimal response to the artificial agent’s actions (level-1 strategy) in all games, as benchmark behavior. In this way, participants’ reliance on the level-2 strategy expressed their level of optimality in the contingent strategic environment. Moreover, since strategies more sophisticated than level-2 (e.g., level-3, Nash equilibrium) are rarely implemented in these games^35–37,40^, the proportion of level-2 choices in the Assessment phase can be considered as a reliable index of participants’ strategic sophistication.

In order to explore participants’ heterogeneity in strategic thinking, we ran a mixture models cluster analysis on proportion of level-2 choices (i.e., responses consistent with the level-2 strategy) in the Assessment phase (See “Statistical data analysis” paragraph in the “Methods” section). Results reveal the presence of three different clusters characterized by increasing levels of strategic sophistication (Low-sophistication: N = 111, mean proportion of level-2 choices = 0.31 ± 0.07; Medium-sophistication: N = 111, mean proportion of level-2 choices = 0.55 ± 0.08; High-sophistication: N = 79, mean proportion of level-2 choices = 0.83 ± 0.08. See Supplementary Figure 1 and Supplementary Table 1 in Supplementary Results). The average level of strategic sophistication of the three clusters is directly linked to their earnings at the end of the Assessment phase (Low-sophistication, mean game payoff (points) = 52.94 ± 4.12, average phase earning (euros) = 4.08 ± 0.32; Medium-sophistication, mean game payoff (points) = 60.35 ± 4.05, average phase earning (euros) = 4.65 ± 0.31; High-sophistication, mean game payoff (points) = 69.52 ± 2.70, average phase earning (euros) = 5.35 ± 0.21).

To characterize participants’ behavior in the three clusters, we computed their relative levels of strategic thinking, as estimated by the CH model. In CH, the frequency distribution f(k) of players’ hierarchical steps of strategic thinking is assumed to be Poisson, and its mean and variance is described by a single parameter τ. The higher the τ of a population, the higher its level of strategic sophistication. Interestingly, players in the Low-sophistication group showed a level of strategic sophistication between level-0 and level-1 (τ = 0.39). Players in the Medium-sophistication cluster were slightly above level-1 (τ = 1.31), in line with the strategy implemented by the artificial agent acting as column player. Eventually, players in the High-sophistication group exhibited choices consistent with the level-2 strategy (τ = 2.16), which represented the best response to the strategy of the counterpart. The level-2 strategy was also implemented with high consistency by all the players selected as the models at the end of the Assessment phase (Mean proportion of level-2 choices = 0.97 ± 0.05, min = 0.82, max = 1). Eleven models (out of 16) showed perfect level-2 behavior and 14 models showed more than 95% of level-2 choices. Players selected as the models were not included in the cluster analysis (See “Statistical data analysis” paragraph in the “Methods” section).

### Investigating the emergence of observational learning

**Figure 2:**
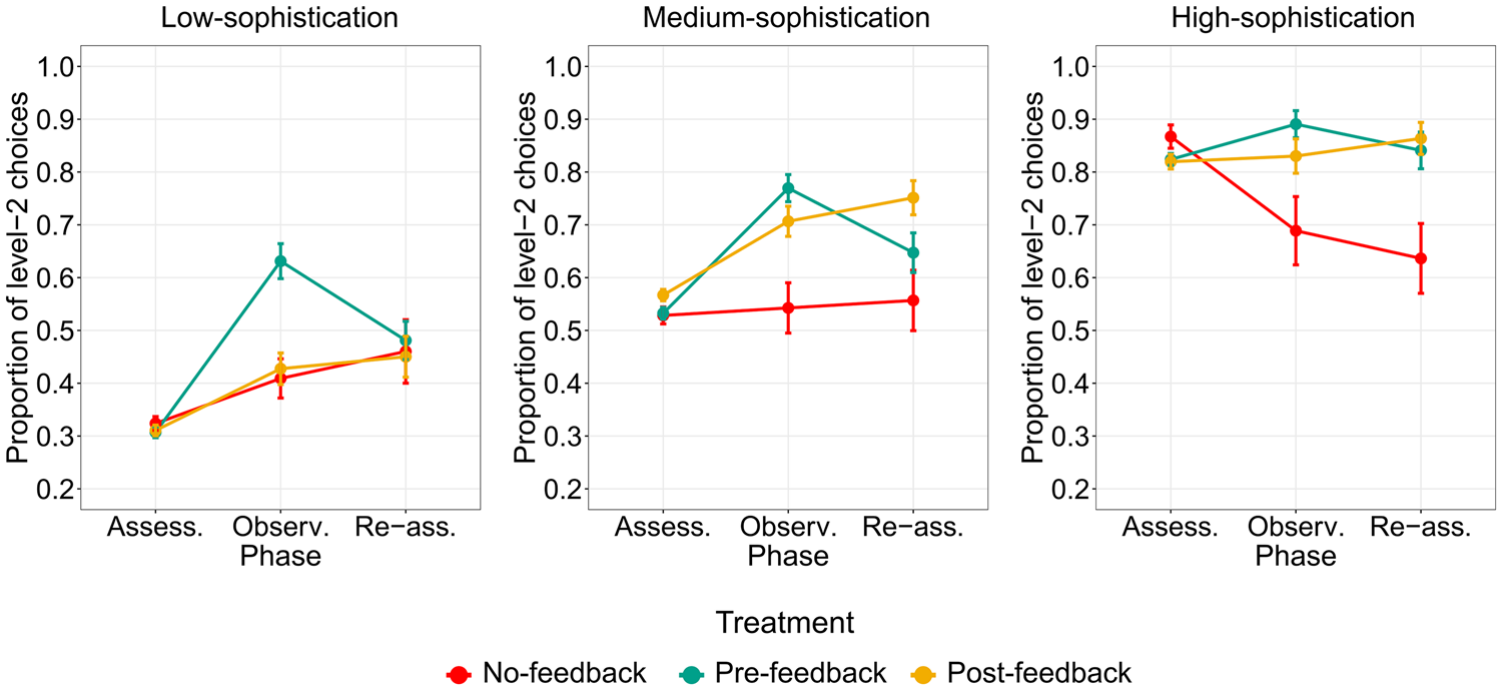
Strategic sophistication across clusters, phases and treatments. Participants were clustered in three groups through a mixture models cluster analysis on the proportion of level-2 choices in the Assessment phase (Low-sophistication, Medium-sophistication and High-sophistication). For each cluster, we plot the proportion of level-2 choices by phase Assessment, Observation and Re-assessment) and treatment (No-feedback, Pre-feedback, Post-feedback). Error bars represent standard errors. In the Medium-sophistication cluster, we show an effect of social learning (Re-assessment–Assessment) in the Post-feedback treatment but not in the Pre-feedback treatment.

We analyzed whether and how participants characterized by different levels of strategic sophistication learned from the model’s behavior observed during the Observation phase (Fig. 2). We investigated whether the timing of the model’s feedback (before participants’ choices in the Pre-feedback treatment, after participants’ choices in the Post-feedback treatment) modulated (1) sophisticated learning or imitation during the Observation phase and (2) transfer of the acquired knowledge in the Re-Assessment phase. In the analysis, results of the Pre-feedback and Post-feedback treatments were always compared to the No-feedback treatment, where we did not provide any feedback on the model’s choices. This aspect is important to specifically investigate the social component of learning that are linked to the observation of the model’s behavior, ruling out potential confounds due to asocial, endogenous learning effects unrelated to learning from the model’s feedback.

### Low-sophistication cluster

We analyzed the behavior of participants in the Low-sophistication cluster using a random effects logistic model with level-2 response as dependent variable, phase (Assessment, Observation, Re-assessment), treatment (No-feedback, Pre-feedback, Post-feedback) and their interactions as independent factors, and a random effect at the subject level (Supplementary Results, Models: Model 1). We used robust standard errors clustered at the subject level (See “Statistical data analysis” paragraph in the “Methods” section).

First, in the No-feedback treatment, we can observe a modest albeit significant learning effect in Observation and Re-Assessment phases, when compared to the Assessment phase (Observation–Assessment 1: regression coefficient (B) = 0.39, z statistic (z) = 2.24, *p* value (p) = 0.025; Re-assessment–Assessment 1: B = 0.61, z = 2.39, *p* = 0.017). This effect could be explained by regression to the mean, given the absence of feedback on game outcomes or model’s choices. Moreover, it may have been amplified by the feedback received at the end of the Assessment phase, who informed participants that they were not the best player(s) in the session, possibly leading to the exploration of alternative strategies in the subsequent phases.

Participants in the Pre-feedback treatment improved their rate of level-2 choices in the Observation phase compared to participants in the No-feedback treatment (interaction effects, Observation–Assessment: B = 1.07, z = 4.59, *p* < 0.001). Nonetheless, the significant improvement in terms of strategic sophistication observed in the Observation phase did not hold in the Re-assessment phase (interaction effects, Re-assessment–Assessment: B = 0.19, z = 0.64, *p* = 0.521). This suggests that participants in the Pre-feedback treatment imitated the model in the Observation phase, but they did not grasp their strategy and were not able to re-apply it in the Re-assessment phase.

Conversely, participants in the Post-feedback treatment did not improve their level of strategic sophistication compared to participants in the No-feedback treatment neither in the Observation phase (interaction effects, Observation–Assessment: B = 0.16, z = 0.69, *p* = 0.488) nor in the Re-assessment phase (interaction effects, Re-assessment–Assessment: B = 0.04, z = 0.12, *p* = 0.908). In the Observation phase, these participants did not have the possibility to imitate the model and were not able to extract information that could improve their own behavioral strategy. Consequently, they did not use a better strategy in the Re-assessment phase. These results suggest that participants starting from a low-strategic level, in the Post-feedback treatment, could not benefit from the model’s feedback in order to improve their behavioral strategy in novel, unguided interactions (Re-assessment phase).

In order to corroborate the results of the Low-sophistication cluster, we estimated the CH model and compared the τ parameter across phases and treatments to investigate changes in strategic sophistication due to social learning (Supplementary Results, Supplementary Table 1). Consistently with the analysis on level-2 choices, we can observe a modest increase in sophistication from the Assessment to the Re-assessment phase, which does not depend on the experimental treatment (Assessment: No-feedback, τ = 0.38; Pre-feedback, τ = 0.38; Post-feedback, τ = 0.39. Re-Assessment: No-feedback, τ = 0.90; Pre-feedback, τ = 0.97; Post-feedback, τ = 0.86). Participants in all treatments, even after observing the model’s feedbacks, slightly increased their strategic level but remained below the level of the artificial agent (level-1) in the Re-assessment phase.

### Medium-sophistication cluster

Then we explored the behavior of participants in the Medium-sophistication cluster by running the same model used for the Low-sophistication group (Supplementary Results, Models: Model 2).

First, we did not found any effect of learning in the No-feedback treatment (Observation–Assessment: B = 0.06, z = 0.33, *p* = 0.744; Re-assessment–Assessment 1: B = 0.12, z = 0.55, *p* = 0.581. Figure 2).

Similarly to the results observed in the Low-sophistication group, participants in the Pre-feedback treatment increased their level of strategic sophistication in the Observation phase compared to participants in the No-feedback treatment (interaction effects, Observation–Assessment: B = 1.10, z = 4.72, *p* < 0.001), whereas learning was absent in the Re-assessment phase (interaction effects, Re-assessment–Assessment: B = 0.40, z = 1.44, *p* = 0.151). This suggests that participants could benefit from imitation in the Observation phase, but they were not able to use the model’s feedback to understand the model’ strategy and improve their own strategic level in the Re-assessment phase. This is particularly interesting if we consider that participants knew that they would face the Re-assessment phase, without feedback from the model, after the Observation phase.

On the contrary, results of participants in the Post-feedback treatment revealed the emergence of sophisticated learning processes (Fig. 2). When compared to the No-feedback treatment, in the Post-feedback treatment medium-sophistication participants were able to learn from the model already in the Observation phase (interaction effects, Observation–Assessment: B = 0.61, z = 2.62, *p* = 0.009), even if they did not have the opportunity to imitate them. Importantly, the improvement in strategic sophistication persisted in the Re-assessment phase (interaction effects, Re-assessment–Assessment: B = 0.80, z = 2.82, *p* = 0.005), suggesting that participants in the Post-feedback treatment implemented a sophisticated social learning process that incorporated the behavioral features underlying the model’s strategy, and therefore succeeded in transferring the acquired knowledge in new interactive environments.

The observed patterns of (social) learning were confirmed by CH model estimation (Supplementary Results, Supplementary Table 1). By comparing τ in Assessment and Re-assessment phases, we can see a decisive increase in sophistication for participants in the Post-feedback treatment, who shifted from level-1 to level-2 behavior (Post-feedback: Assessment, τ = 1.34; Re-assessment, τ = 2.06). Conversely, participants in No-feedback and Post-feedback treatments slightly increased their strategic level but maintained a sophistication level between level-1 and level-2 (No-feedback: Assessment, τ = 1.15; Re-assessment, τ = 1.50; Pre-feedback: Assessment, τ = 1.34; Re-assessment, τ = 1.77).

**Figure 3:**
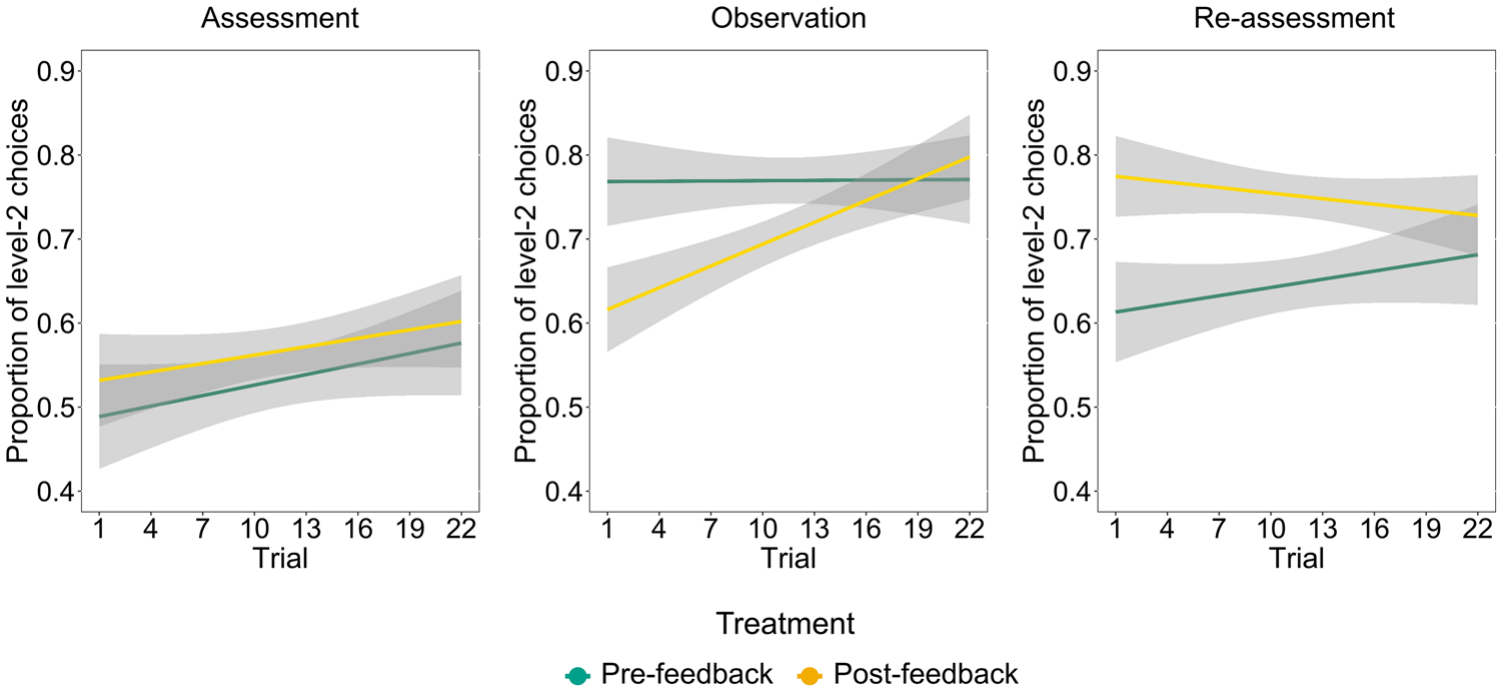
Temporal evolution of strategic sophistication across phases in the Medium-sophistication cluster. We characterize the learning process of participants in the Medium-sophistication cluster, in which we observed an effect of social learning, by plotting the temporal evolution of the proportion of level-2 choices in Pre-feedback and Post-feedback treatments. Trial, ordered by time of presentation, has been used as time variable. Grey bounds represent standard errors. Data has been smoothed by linear fit. Results reveal a significant dynamic learning effect along the Observation phase for participants in the Post-feedback treatment, but not for participants in the Pre-feedback treatment.

We further explored the different social learning effects exerted by the model’s feedback in Post-feedback and Pre-feedback treatments by observing the temporal evolution of sophistication along the Observation phase, during accumulation of information from the model (Fig. 3). We ran a random effects logistic model (Supplementary Results, Models: Model 3) with level-2 response in the Observation phase as dependent variable, (social) treatment (Post-feedback and Pre-feedback), trial order and their interaction as independent variables. Results reveal a significant interaction effect (Post-feedback–Pre-feedback: B =−0.05, z =−2.40, *p* = 0.016) that was explained by a significant effect of trial order in the Post-feedback treatment (B = 0.05, z = 3.60, *p* < 0.001) but not in the Pre-feedback one (B = 0.00, z = 0.05, *p* = 0.959). As expected, these learning effects were absent in Assessment and Re-assessment phases, when participants did not receive any feedback from the model (Supplementary Results, Models: Model 3). The findings of the temporal analysis confirm that participants in the Post-feedback treatment were able to accumulate useful information from the model’s choices, leading them to increase their level of strategic sophistication along the Observation phase. As also pointed out in the previous analysis, this learning process led participants in the Post-feedback treatment to maintain a high level of strategic sophistication in the Re-assessment phase, in contrast to participants in the Pre-feedback treatment.

### High-sophistication cluster

Eventually, we analyzed the behavior of participants in the High-sophistication cluster using the same analysis of the other two clusters (Supplementary Results, Models: Model 4).

First, results of the No-feedback treatment suggest a significant decline in sophistication in Observation and Re-assessment phases (Observation–Assessment: B =−1.19, z =−3.40, *p* = 0.001; Re-assessment–Assessment: B =−1.46, z =−4.94, *p* < 0.001). One the one hand, this effect could be explained by the feedback provided at the end of the Assessment phase. Indeed, participants were informed that they were not the best player in the session (even if their performance was high), potentially leading to detrimental strategy switches in absence of proper feedback about the best player’s strategy. On the other hand, the observed decline might be due to simple regression to the mean, always related to the absence of precise feedback about their general and game-by-game performance.

The effect of performance decay in the No-feedback treatment led to a relatively higher rate of level-2 responses in both Post-feedback and Pre-feedback treatments in Observation and Re-assessment phases (Interaction effects. Pre-feedback, Observation–Assessment: B = 1.78, z = 4.09, *p* < 0.001; Re-assessment–Assessment: B = 1.59, z = 3.89, *p* < 0.001; Post-feedback, Observation–Assessment: B = 1.27, z = 2.95, *p* = 0.003; Re-assessment–Assessment: B = 1.83, z = 4.61, *p* < 0.001). Taking apart the performance decay observed in the No-feedback treatment, we did not find any learning effect in the Re-assessment phase in neither Pre-feedback nor Post-feedback treatments (Pre-feedback, Re-assessment–Assessment: B = 0.13, z = 0.46, *p* = 0.645; Post-feedback, Re-assessment–Assessment: B = 0.37, z = 1.37, *p* = 0.169), likely due to ceiling effects.

These effects were confirmed by results of the CH model estimation. The τ parameter slightly decreased in the No-feedback treatment (No-feedback: Assessment, τ = 2.23; Re-assessment, τ = 1.87), whereas the strategic level of participants in Pre-feedback and Post-feedback conditions did not change considerably between phases (Pre-feedback: Assessment, τ = 2.14; Re-assessment, τ = 2.25; Post-feedback: Assessment, τ = 2.16; Re-assessment, τ = 2.29).

## Results summary

Altogether, our results reveal that observational learning led to an improvement in strategic thinking only in specific circumstances, depending on participants’ initial level of strategic sophistication and the timing of social feedback. Participants showing an initial low strategic level (Low-sophistication cluster) were not able to learn from the model and transfer the observed sophisticated behavior to novel interactive scenarios. Indeed, they merely imitated the model when they had the opportunity to do so (Pre-feedback treatment) but did not grasp the rationale underlying the model’s behavior and did not apply the observed strategy in the Re-assessment phase in any of the experimental treatments. Players in the Medium-sophistication cluster succeeded in increasing their level of sophistication in the Re-assessment phase, but only when they did not have the possibility to imitate blindly the model’s choices (Post-feedback treatment). Conversely, participants in the Pre-feedback treatment imitated the model’s choices during observation, but were not able to re-apply this strategy in the Re-assessment phase. Eventually, participants in the High-sophistication cluster benefited from the observation of the model (Pre- and Post-feedback treatments), since they managed to maintain a high level of strategic thinking throughout the entire experiment, in contrast with participants in the No-feedback treatment, whose performance dropped significantly in the Re-assessment phase.

## Discussion

The current study offers novel evidence on how and under what circumstances individuals use observational learning to enhance their level of strategic sophistication during social interaction. Our results can be summarized in three main points:

1. Players with a low initial level of strategic sophistication do not succeed in learning from observation and transferring the acquired knowledge to novel, unguided contexts.
2. Players with a sufficient initial level of strategic thinking learn through observation only if they do not have the possibility to imitate the behavioral model; if they can imitate, they do it blindly and do not generalize the acquired knowledge to new games.
3. Players with a high initial level of strategic thinking use social feedback to maintain optimal behavior even in the absence of precise feedback on their performance.

The observed inability of unsophisticated players to learn from the observation of a (sophisticated) model may be linked to insufficient cognitive abilities. Extensive evidence has shown that strategic sophistication in interactive games positively correlates with fluid intelligence^32,33,41,42^ and cognitive reflection^30,31,35,43–46^. Indeed, unsophisticated participants may have failed in grasping the fundamental features of the interactive scenarios and the model’s strategy, preventing sophisticated learning and generalization in new games. Another possible explanation for the absence of a learning effect in low-sophistication participants lies in the marked discrepancy from the observed model’s behavior, which may have complicated the understanding of the rationale behind the model’s choices, as generally observed in multi-agent systems^47^. Moreover, a high distance from an (overtly) efficient behavior could have decreased participants’ confidence, a condition that generally promotes effortless imitative behavior^48^. These findings offer new insights in the understanding of whether and when individuals can enhance their level of strategic thinking when exposed to different behavioral strategies^49,50^.

Moreover, we have shown that players with an adequate (but not optimal) strategic level learn from observation only if they do not have the opportunity to imitate blindly the behavioral model. This finding suggests that the learning context plays an important role in guiding the selection of different social learning strategies in interactive scenarios, which is generally modulated by the interplay between the benefits and the relative cognitive costs of the available strategies^9,21,51,52^. Our findings complement theoretical, empirical and computational models of social learning describing the adaptive role of asocial and social learning strategies based on their respective cost and efficiency in non-interactive contexts^8,18,22,53,54^. Interestingly, recent neuro-computational findings^20^ revealed that a brain network including ventrolateral prefrontal cortex, temporoparietal junction and rostral cingulate cortex dynamically represents the current reliability of sophisticated (i.e., emulative) learning strategies, signaling the circumstances under which it may be beneficial to select it over simple imitation. These findings, in line with well-established models of social learning^9,21,52^, suggest that imitation is a sort of default strategy, which is implemented whenever more sophisticated learning processes are too costly or their benefit is uncertain. In accordance with this idea, we show that fundamental exogenous characteristics of the social feedback (i.e. whether it is provided before or after agents’ choices) can determine the degree of reliance on either imitative or sophisticated learning strategies by modulating the cost/benefit ratio associated with them. In particular, we suggest that receiving social feedback before our choices can naturally bias our process or arbitration towards (blind) imitation, whereas receiving social feedback after our choices promotes the implementation of more sophisticated learning strategies. Our results enrich models of social learning describing the so-called “When” strategies, which indicate the circumstances under which individuals imitate others^11^. We hope our study to fuel further research on the role of the informational context and the timing of social feedback in the emergence of different forms of social learning also in non-interactive settings.

Furthermore, the current findings highlight that providing social feedback before agents’ choices my lead to detrimental consequences in terms of long-term learning and generalization. Participants in the Pre-feedback treatment could highly benefit from imitative behavior in the Observation phase with very low cost, which led them to sacrifice the long-term benefits of sophisticated, structured learning in the Re-assessment phase. This was the case even if participants knew from the beginning of the experiment that they would perform a final phase without the model’s feedback. We suggest that providing a costless behavioral shortcut such as blind imitation to individuals may discourage the implementation of sophisticated forms of social learning, preventing transfer of the acquired knowledge to novel contexts. Our study offers novel insights on the limitations and drawbacks of imitation, complementing previous theoretical accounts and experimental studies in economics^55,56^. Eventually, results of the cluster including highly sophisticated participants revealed that social feedback from an efficient behavioral model is important for the preservation of adaptive behavior. Indeed, in the absence of exogenous feedback revealing precise choice outcomes, sophisticated individuals may be tempted to explore alternative, inefficient behaviors. Conversely, if sophisticated actors receive feedback from an agent who is known to be successful and show a behavior similar to theirs, they can positively reinforce their strategy. This is in line with research highlighting the benefits arising from the gathering of different opinions in multi-agent systems^57–61^.

This is one of the first studies investigating social observational learning in interactive contexts. Our results are crucial in the comprehension of how social and informational contexts may bias the endogenous process of selection of learning strategies, offering novel insights for theoretical models of social learning, cultural evolution and strategic interaction. Crucially, our findings may have important applications in educational contexts, where instrumental feedback from instructors should be calibrated to discourage unsophisticated and simplistic learning strategies and promote sophisticated learning processes that can guarantee knowledge generalizability. By further investigating the interplay between informational context and social learning mechanisms, we will be able to understand the factors that can encourage the development of sophisticated learning processes in human beings of different ages, which may determine the success of the educational programs promoted by human organizations.

## Methods

### Overview: participants and procedure

Three hundred twenty one (321) students from the University of Trento participated in our study (175 females, mean age: 21.16, SD: 2.01). Participants with a formal knowledge of game theory were excluded through a preliminary screening. Moreover, we tested participants from different departments of the University of Trento to guarantee that our sample is representative of a typical (student) population.

The experimental task was carried out in 16 separate between-subject sessions. The average number of participants in each of the sessions was 20.06 ± 2.84 (min 15, max 24). Each session was associated with one of three experimental treatments: Pre-feedback, Post-feedback and No-feedback. Pre-feedback and Post-feedback treatments consisted of 7 sessions each (Pre-feedback, N = 136; Post-feedback, N = 139), whereas 2 sessions (N = 47) were assigned to the No-feedback treatment, which served as control treatment. All participants, independently of the experimental treatment, completed the entire experimental paradigm, which included three consecutive experimental phases always presented in the same order (Assessment, Observation, Re-assessment).

The experiment was conducted at the Experimental Economics Laboratory at the University of Trento (CEEL). The experimental room included 24 different computers connected with a central computer and the main server through z-Tree/z-Leaf. All participants assigned to a given session were asked to come to the lab at a given time. They were randomly assigned with a number, which corresponded to the number of one of the computers in the room. Each participant was invited to sit in front of the relative computer. Each computer was surrounded by portioning panels preventing participants to see others’ computers. Each participant received a printed copy of the instructions, which were also read aloud by one of the experimenters. In this phase, participants could ask questions. Moreover, participants had to complete a comprehension test to investigate if they had understood the experimental task (Supplementary Information, Comprehension test). We checked the participants' answers before starting the experiment and provided additional explanations in the case of mistakes or doubts. During the experiment, participants were asked not to communicate with each other or making comments aloud. In each session, three experimenters were present (in different parts of the room) to ensure the correct execution of the experimental session and ensure that participants did not talk to each other or take notes about what they were doing. If participants had a question during the experiment (e.g., between the experimental phases), they were asked to raise their hand and talk privately with one of the experimenters.

In the Assessment phase, participants in all the three treatments played 22 one-shot 3 × 3 matrix games with an artificial agent (see “Games and artificial agent behavior” paragraph in the “Methods” section). Participants played as the row players and had to choose one of three possible actions, represented by the three rows of the matrix (Fig. 1b). Conversely, the artificial agent chose one of the three columns of the game matrix. The combination of both players’ choices determined an outcome for each of them. Participants selected an action through mouse click and could change their choice until they pressed the “confirm” button.

At the end of the Assessment phase, in all treatments, each participant was told if they were the best player in the session (i.e., the “model”), based on the cumulative score of the obtained payoffs in the 22 games (Fig. 1c). If two or more participants accumulated the same best score in a given session, each of them was told to be the best player in the session and one of them was randomly selected as the model for the subsequent experimental phase (Observation phase). Anyway, we acknowledge that participants that accumulated the same cumulative score made identical choices.

Then participants underwent the Observation phase, in which they played again the same 22 games with the same artificial agent. The order of the 22 games was randomized. Nevertheless, participants in Pre-feedback and Post-feedback treatments could receive feedback about the choice taken *in the Assessment phase* by the mode in that game. The type of feedback received by participants in the Observation phase depended on the experimental treatment. In the Pre-feedback treatment, participants received a feedback on the decision taken by the model in that specific game as soon as the game matrix had appeared, *before* they made their decision. Conversely, participants in the Post-feedback treatment received the model’s feedback only *after* they made their decision, which could not be changed based on this feedback. The model’s feedback consisted in a black arrow pointing at the row selected by the model in the Assessment phase (Fig. 1b). In the No-feedback treatment, participants did not receive any feedback on the models’ choices. Participants in all the treatments did not receive any other feedback about the game, including the choice made by the artificial agent and the game outcome.

Eventually, participants in all the treatments underwent the Re-assessment phase, in which they played 22 new games without any feedback from the model, as in the Assessment phase (see “Games and artificial agent behavior” paragraph in the “Methods” section). A detailed and exhaustive explanation of all the three phases was provided to participants before starting the entire experimental session. Therefore, in every experimental phase, participants were aware of the subsequent phases. Moreover, at the end of each experimental phase, participants were reminded of the upcoming phases. Participants’ reimbursement was calculated based on their cumulative score in the 66 games played across the three experimental phases. In particular, the sum of the points accumulated during the entire experiment was multiplied by 0.0035 to obtain an amount varying between 6 and 17 euros. Moreover, we added 3 euros of show-up fee to this variable amount to obtain the final participants’ reimbursement (min. 9, max. 20 euros). The study was approved by the Human Research Ethics Committee of the University of Trento (protocol title: “Transfer learning within and between brains”). All participants gave informed consent. All experiments were performed in accordance with the relevant guidelines and regulations.

### Games and artificial agent behavior

Participants in all treatments played, as row player, 22 one-shot 3 × 3 matrix games in Assessment and Observation phases, and 22 new one-shot 3 × 3 matrix games in the Re-assessment phases. In the Supplementary Information (Supplementary Methods, Supplementary Figure 2), we report the entire list of games used in the three phases. The games used in the Re-assessment phase maintained the same payoff structure of the games used for Assessment and Observation phases, although the actual payoffs were different. Specifically, in each game payoffs were changed by adding or subtracting a small constant; moreover, the order of the three actions was swapped. We included 10 dominance-solvable games, solvable with different steps of iterated dominance, and 12 games with unique Nash equilibrium without dominant solution. All games have a unique Nash equilibrium in pure strategies. This guarantees that the Nash equilibrium solution corresponds to the highest level of strategic thinking, as estimated by the Cognitive Hierarchy (CH) model.

The artificial agent acting as the column player played with a fixed strategy, which was unknown to participants. However, they were told that the agent would use the most common strategy in those games. The selection of the artificial agent strategy followed the behavioral characterization of models of strategic thinking such as CH. In particular, the agent implemented the level-1 strategy, which consisted in the selection of the column with the highest average payoff for itself, independently on the potential actions of the counterpart. Indeed, recent studies revealed that the level-1 strategy is the most common strategy in similar 3 × 3 one-shot games^35–37^. These studies also suggest that the Nash equilibrium is rarely played in this kind of games and that the maximum level of strategic sophistication implemented by participants is generally level-2.

Thus, the best strategy in all games was the level-2 strategy and not the Nash equilibrium. Supplementary Figure 2 (Supplementary Methods) reports, for each game, the Nash equilibrium, the column selected by the artificial agent (level-1 strategy) and the row representing the participants’ best-response (level-2 strategy).

### Statistical data analysis

The main analyses, aimed at investigating social learning in strategic interaction, were run excluding participants selected as models at the end of the Assessment phase, since they were told to be the best player in their session and observed their own choices in the Observation phase. In case of a tie in a specific session, all the participants scoring the best equal score were excluded. Therefore, the main analyses, including the cluster analysis, were run on a sample of 301 participants. First, we classified participants in different groups based on their initial level of strategic sophistication, by looking at choice behavior in the Assessment phase. Responses were classified as either consistent or inconsistent with the level-2 strategy, which expressed the best response to the strategy used by the artificial agent acting as the participants’ counterpart in the games (level-1). Therefore, we ran a mixture models cluster analysis^62,63^ on proportion of level-2 choices in the Assessment phase. The main advantage of this clustering method is that we did not need to assume a priori neither the number of clusters nor the clustering criterion. Analyses on social learning were run separately for each strategic level cluster, as defined by the mixture model cluster analysis. Within each cluster, we analyzed the effect of learning across phases (Assessment, Observation, Re-assessment) and treatments (No-feedback, Pre-feedback and Post-feedback). We used random effects logistic models with level-2 choice (1: consistent with the level-2 strategy; 0: inconsistent with the level-2 strategy) as binary dependent variable and random effect at the subject level. Random effects were applied to the intercept to adjust for the baseline level of sophistication of each subject and model intra-subject correlation of repeated measurements. The variance–covariance matrix of all models was estimated using robust variance estimator^64–66^ to obtain heteroscedasticity-robust standard errors clustered at the subject level. In the Results section of the main text, we report regression coefficients (B), test statistics (z) and *p* values for random effects models. In the Supplementary Information (Supplementary Results), we report complete fixed-effects results including robust standard errors and confidence intervals.

We acknowledge that level-2 choices were strongly correlated with game payoffs, given that the computer behavior was constant along the experiment, and that level-2 response was always the best-response to this strategy. Specifically, participants’ proportion of level-2 choices (along the entire experiment) show an almost perfect correlation (r = 0.96, *p* < 0.001) with participants’ average game outcome. For this reason, we generally reported results using level-2 response as dependent variable, although results are comparable using participants’ game outcomes as dependent variable.

To better characterize the evolution of strategic sophistication along the experiment, we also estimated the level of strategic thinking predicted by the Cognitive Hierarchy (CH) model across clusters, treatments and phases. In CH, the frequency distribution f(k) of steps of players is assumed to be Poisson, and its mean and variance is described by a single parameter τ. The higher the τ of a population, the higher its level of strategic sophistication. By analyzing the temporal evolution (i.e., across phases) of the τ parameter for each cluster in the different treatments, we could explore the emergence of (social) learning effects underlying the increase (or decrease) of participants’ strategic sophistication.

## Supplementary Information

Supplementary Information.

## Data Availability

The datasets supporting all the analyses, findings and figures included in the current study are available in a dedicated OSF repository at: https://osf.io/gkj9m/?view_only=a847c3d73eaa41cd9f3c8a35fafc6c07.

## Code availability

The scripts used for collecting data and generating results during the current study are available from the corresponding author upon request.
