# Supplementary material for: Timing of social feedback shapes observational learning in strategic interaction
Source: Sci Rep. 2021 Nov 9;11:21972. doi: 10.1038/s41598-021-01466-1 (PMC8578421; doi:10.1038/s41598-021-01466-1)
Supplement: Supplementary file 1 — Supplementary Information. [file 41598_2021_1466_MOESM1_ESM.pdf]

Supplementary Information

Timing of social feedback shapes observational learning in strategic interaction

Joshua Zonca*[1], Alexander Vostroknutov[2], Giorgio Coricelli[3], Luca Polonio[4]

1. Cognitive Architecture for Collaborative Technologies (CONTACT) Unit, Italian Institute of Technology, Genoa, Italy.

2. Department of Economics (MPE), Maastricht University, Maastricht, The Netherlands.

3. Department of Economics, University of Southern California, Los Angeles, USA.

4. Molecular Mind Laboratory (MoMiLab) Unit, IMT School for Advanced Studies Lucca, Lucca, Italy.

Supplementary Results

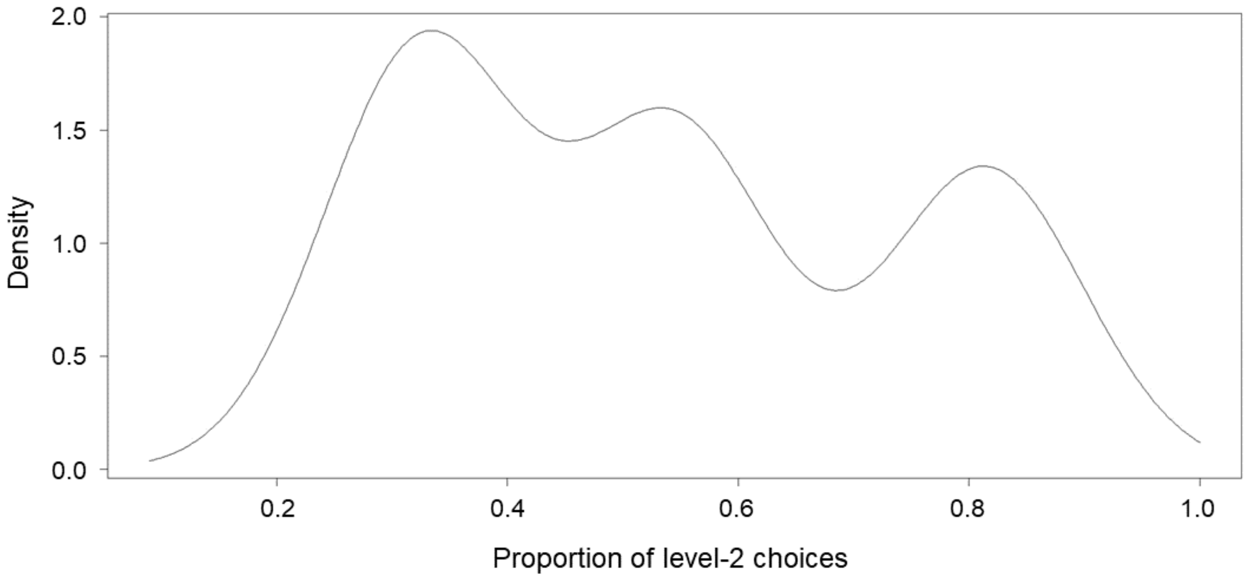

Supplementary Figure 1. Density plot representing the distribution of participants' levels of strategic sophistication (i.e., individual proportions of level-2 choices) in the Assessment phase. Looking at the distribution, we can appreciate the presence of three clusters of players, in line with the results of the mixture models cluster analysis (See Supplementary Table 1).

		Assessment		Re-assessment	
Cluster / Treatment	N	τ (CH)	Proportion of level-2 choices	τ (CH)	Proportion of level-2 choices
Low-sophistication					
No-feedback	16	0.38	0.32 (0.05)	0.90	0.46 (0.24)
Pre-feedback	53	0.38	0.31 (0.07)	0.97	0.48 (0.26)
Post-feedback	42	0.39	0.31 (0.07)	0.86	0.45 (0.25)
All	111	0.39	0.31 (0.07)	0.91	0.47 (0.25)
Medium-sophistication					
No-feedback	16	1.15	0.53 (0.06)	1.50	0.56 (0.23)
Pre-feedback	42	1.34	0.53 (0.07)	1.77	0.65 (0.24)
Post-feedback	53	1.34	0.57 (0.08)	2.06	0.75 (0.23)
All	111	1.31	0.55 (0.08)	2.00	0.68 (0.25)
High-sophistication					
No-feedback	13	2.23	0.87 (0.08)	1.87	0.64 (0.24)
Pre-feedback	32	2.14	0.82 (0.06)	2.25	0.84 (0.20)
Post-feedback	34	2.16	0.82 (0.08)	2.29	0.86 (0.18)
All	79	2.16	0.83 (0.08)	2.25	0.82 (0.21)

Supplementary Table 1. Participants were classified in three clusters of increasing strategic sophistication through a mixture models cluster analysis on individuals' proportion of level-2 choices. We report the parameter τ (CH), which expresses the average group level of strategic thinking in the Cognitive Hierarchy (CH) model, and the average proportion of level-2 responses (standard deviations in brackets) across clusters (Low-sophistication, Medium-sophistication, High-sophistication), treatments (No-feedback, Pre-feedback, Post-feedback) and phases (Assessment, Re-assessment).

Models

We report the details of the random effects models ran in the present work. For all models**,** the intercept was allowed to vary across participants including random effects at subject level. We estimated the variance-covariance matrix of all models using robust variance estimator to obtain heteroscedasticity-robust stand errors clustered at the subject level.

In every model equation, β expresses coefficients of fixed effects, while u indicates random effects.

In the fixed-effect results, B express (unstandardized) regression coefficients. Independent categorical variables (*Treatment* and *Phase*, 3 levels) have been treated as dummy variables.

Model 1: Effects of treatment by phase on level-2 choices: Low-sophistication cluster

In Model 1, we consider only participants clustered in the Low-sophistication group. We tested the effects of treatment (No-feedback, Pre-feedback, Post-feedback) and phase (Assessment, Observation, Re-assessment), along as their interactions, on participants' strategic (level-2) choices (binary outcome variable: 1 = consistent with level-2 strategy; 0 = inconsistent with level-2 strategy).

We used the following random effects model:

$$L = \beta_0 + \beta_1 t + \beta_2 p + \beta_3 t * p + u_0 + \varepsilon$$

L is the participants' (level-2) choice, t is a dummy variable for treatment and p is a dummy variable for phase. No-feedback and Assessment are the reference levels for treatment and phase factors, respectively.

Results:

Level-2 choice	B	Robust Std. Err.	z	p	95% Conf. Inter.	
Treatment (Assessment phase)						
Pre-feedback – No-feedback	- 0.108	0.085	- 1.26	0.206	- 0.275	0.059
Post-feedback – No-feedback	- 0.077	0.084	- 0.91	0.361	- 0.241	0.088
Phase (Baseline treatment)						
Observation – Assessment	0.388	0.173	2.24	0.025	0.049	0.727
Re-assessment – Assessment	0.608	0.254	2.39	0.017	0.110	1.105
Treatment*Phase						
Observation – Assessment (Pre-feedback – No-feedback)	1.075	0.234	4.59	< 0.001	0.615	1.534
Re-assessment – Assessment (Pre-feedback – No-feedback)	0.191	0.297	0.64	0.521	- 0.392	0.773
Observation – Assessment (Post-feedback – No-feedback)	0.156	0.225	0.69	0.488	- 0.285	0.597
Re-assessment – Assessment (Post-feedback – No-feedback)	0.036	0.310	0.12	0.908	- 0.572	0.644
N. obs	7326					
N. groups	111					

Model 2: Effects of treatment by phase on level-2 choices: Medium-sophistication cluster

In Model 2, we consider only participants clustered in the Medium-sophistication group. We tested the effects of treatment (No-feedback, Pre-feedback, Post-feedback) and phase (Assessment, Observation, Re-assessment), along as their interactions, on participants' strategic (level-2) choices (binary outcome variable: 1 = consistent with level-2 strategy; 0 = inconsistent with level-2 strategy).

We used the following random effects model:

$$L = \beta_0 + \beta_1 t + \beta_2 p + \beta_3 t * p + u_0 + \varepsilon$$

L is the participants' (level-2) choice, t is a dummy variable for treatment and p is a dummy variable for phase. No-feedback and Assessment are the reference levels for treatment and phase factors, respectively.

Results:

Level-2 choice	B	Robust Std. Err.	z	p	95% Conf. Inter.	
Treatment (Phase 1)						
Pre-feedback – No-feedback	0.013	0.086	0.15	0.883	- 0.156	0.181
Post-feedback – No-feedback	0.162	0.086	1.89	0.059	- 0.006	0.330
Phase (Baseline treatment)						
Observation – Assessment	0.062	0.189	0.33	0.744	- 0.308	0.431
Re-assessment – Assessment	0.123	0.224	0.55	0.581	- 0.315	0.562
Treatment*Phase						
Observation – Assessment (Pre-feedback – No-feedback)	1.104	0.234	4.72	< 0.001	0.646	1.562
Re-assessment – Assessment (Pre-feedback – No-feedback)	0.397	0.277	1.44	0.151	- 0.145	0.940
Observation – Assessment (Post-feedback – No-feedback)	0.614	0.235	2.62	0.009	0.155	1.074
Re-assessment – Assessment (Post-feedback – No-feedback)	0.800	0.283	2.82	0.005	0.245	1.356
N. obs	7326					
N. groups	111					

Model 3: Effects of treatment by trial on level-2 choices for the Medium-sophistication cluster in the Observation phase

In Model 3, we further explored the dynamics of the learning effects in Pre-feedback and Post-feedback treatments observed in Model 2. We consider only data of the Medium-sophistication group and choices implemented in the Observation phase. We tested the effects of (social) treatment (Pre-feedback, Post-feedback) and trial, along as their interaction, on participants' strategic (level-2) choices (binary outcome variable: 1 = consistent with level-2 strategy; 0 = inconsistent with level-2 strategy). We used the following random effects model:

$$L = \beta_0 + \beta_1 t + \beta_2 o + \beta_3 t * o + u_0 + \varepsilon$$

L is the participants' (level-2) choice, t is a dummy variable for treatment and o is a continuous variable for the current trial, ordered based on the time of presentation during the Observation phase. The Post-feedback phase is the reference level for the treatment factor.

Results:

Level-2 choice	B	Robust Std. Err.	z	p	95% Conf. Inter.	
Treatment (Pre-feedback – Post-feedback)	0.928	0.319	2.91	0.004	0.304	1.552
Trial	0.051	0.014	3.60	< 0.001	0.023	0.079
Treatment*Trial (Pre-feedback – Post-feedback)	- 0.050	0.021	- 2.40	0.016	- 0.091	- 0.009
N. obs	2090					
N. groups	95					

We replicated the same analysis for Assessment and Re-assessment phase and, as expected, we did not find any dynamic learning effect, since participants did not receive any feedback from the model. In the Assessment phase, results reveal no interaction effect between treatment and trial order (Post-feedback - Pre-feedback: B = - 0.00, z = - 0.18, p = 0.855) and no effect of trial neither in the Post-feedback treatment (B = 0.01, z = 1.01, p = 0.314) nor in the Pre-feedback one (B = 0.02, z = 1.55, p = 0.122). Similarly, in the Re-assessment phase results reveal no significant interaction (Post-feedback - Pre-feedback: B = - 0.03, z = - 1.93, p = 0.053) and no effect of trial neither in the Post-feedback treatment (B = - 0.02, z = - 1.25, p = 0.211) nor in the Pre-feedback one (B = 0.02, z = 1.48, p = 0.138).

Model 4: Effects of treatment by phase on level-2 choices: High-sophistication cluster

In Model 4, we consider only participants clustered in the High-sophistication group. We tested the effects of treatment (No-feedback, Pre-feedback, Post-feedback) and phase (Assessment, Observation, Re-assessment), along as their interactions, on participants' strategic (level-2) choices (binary outcome variable: 1 = consistent with level-2 strategy; 0 = inconsistent with level-2 strategy).

We used the following random effects model:

$$L = \beta_0 + \beta_1 t + \beta_2 p + \beta_3 t * p + u_0 + \varepsilon$$

L is the participants' (level-2) choice, t is a dummy variable for treatment and p is a dummy variable for phase. No-feedback and Assessment are the reference levels for treatment and phase factors, respectively.

Results:

Level-2 choice	B	Robust Std. Err.	z	p	95% Conf. Inter.	
Treatment (Phase 1)						
Pre-feedback – No-feedback	- 0.436	0.253	- 1.72	0.086	- 0.923	0.061
Post-feedback – No-feedback	- 0.413	0.262	- 1.58	0.115	- 0.926	0.100
Phase (Baseline treatment)						
Observation – Assessment	- 1.191	0.351	- 3.40	0.001	- 1.878	- 0.504
Re-assessment – Assessment	- 1.459	0.295	- 4.94	< 0.001	- 2.038	- 0.880
Treatment*Phase						
Observation – Assessment (Pre-feedback – No-feedback)	1.779	0.435	4.09	< 0.001	0.926	2.633
Re-assessment – Assessment (Pre-feedback – No-feedback)	1.590	0.409	3.89	< 0.001	0.789	2.390
Observation – Assessment (Post-feedback – No-feedback)	1.274	0.432	2.95	0.003	0.428	2.120
Re-assessment – Assessment (Post-feedback – No-feedback)	1.827	0.397	4.61	< 0.001	1.050	2.604
N. obs	5214					
N. groups	79					

Supplementary Methods

Assessment & Observation phases

Game 1	i	ii	iii
I	78,73	69,23	12,14
II	60,52	59,66	78,53
III	16,76	65,82	94,79

Game 2	i	ii	iii
I	21,67	64,57	80,63
II	71,76	50,58	74,14
III	12,10	51,76	77,92

Game 3	i	ii	iii
I	74,38	58,71	66,43
II	96,12	10,89	57,25
III	15,51	83,18	69,62

Game 4	i	ii	iii
I	73,60	20,85	91,12
II	45,68	64,71	27,59
III	40,76	53,17	14,98

Game 5	i	ii	iii
I	78,49	60,68	27,35
II	10,82	49,10	97,38
III	59,64	52,39	85,56

Game 6	i	ii	iii
I	39,98	36,28	57,86
II	83,11	50,79	65,60
III	11,50	69,61	40,53

Game 7	i	ii	iii
I	84,82	33,95	12,73
II	21,28	39,37	68,64
III	70,39	59,48	31,81

Game 8	i	ii	iii
I	47,30	94,32	36,38
II	38,69	81,83	27,20
III	80,58	72,11	63,37

Game 9	i	ii	iii
I	46,58	57,34	74,70
II	89,32	31,83	12,41
III	41,94	16,37	53,23

Game 10	i	ii	iii
I	34,59	60.91	96,43
II	36,48	85,33	39,18
III	72,76	43,14	25,55

Game 11	i	ii	iii
I	43,91	38,81	92,64
II	39,27	79,68	68,19
III	69,10	66,21	74,54

Game 12	i	ii	iii
I	25,27	90,43	38,60
II	49,39	53,73	78,52
III	64,85	20,46	19,78

Game 13	i	ii	iii
I	83,40	23,68	70,81
II	93,45	12,71	29,41
III	56,94	66,76	21,70

Game 14	i	ii	iii
I	82,61	36,46	24,22
II	43,17	70,50	40,87
III	75,16	49,75	57,35

Game 15	i	ii	iii
I	40,71	95,34	47,82
II	22,66	66,55	76,35
III	63,50	30,14	54,46

Game 16	i	ii	iii
I	36,97	57,68	16,32
II	84,49	37,78	48,56
III	73,42	68,24	52,65

Game 17	i	ii	iii
I	13,38	69,65	59,70
II	55,65	18,72	64,83
III	90,51	42,67	46,30

Game 18	i	ii	iii
I	36,11	63,53	49,88
II	45,67	70,16	65,40
III	48,57	81,62	28,44

Game 19	i	ii	iii
I	97,72	60,33	43,84
II	13,25	65,57	84,50
III	43,37	83,59	14,54

Game 20	i	ii	iii
I	86,45	52,86	56,16
II	74,99	27,15	39,45
III	35,62	59,67	61,85

Game 21	i	ii	iii
I	68,50	25,14	54,46
II	19,73	69,55	72,38
III	39,60	94,33	48,79

Game 22	i	ii	iii
I	48,56	40,74	81,50
II	52,70	75,21	62,41
III	16,27	57,71	35,96

Re-assessment phase

Game 1	i	ii	iii
I	63,80	14,75	92,77
II	67,21	76,71	10,12
III	57,65	58,50	76,51

Game 2	i	ii	iii
I	14,12	53,78	79,94
II	23,69	66,59	82,65
III	73,38	52,60	76,16

Game 3	i	ii	iii
I	99,15	13,92	60,28
II	18,54	86,21	72,65
III	77,41	61,74	69,46

Game 4	i	ii	iii
I	12,96	38,74	51,15
II	89,10	71,58	18,83
III	25,57	43,66	62,69

Game 5	i	ii	iii
I	99,40	51,12	12,84
II	87,58	54,41	61,66
III	29,37	62,70	80,51

Game 6	i	ii	iii
I	70,62	41,54	12,51
II	37,29	58,87	40,99
III	51,80	66,61	84,12

Game 7	i	ii	iii
I	66,62	19,26	37,35
II	29,79	68,37	57,46
III	10,71	82,80	31,93

Game 8	i	ii	iii
I	80,82	37,68	26,19
II	93,31	46,29	35,37
III	71,10	79,57	62,66

Game 9	i	ii	iii
I	11,40	88,31	30,82
II	52,22	40,93	15,36
III	73,69	45,57	56,33

Game 10	i	ii	iii
I	22,52	69,73	40,11
II	93,40	31,56	57,88
III	36,15	33,45	82,30

Game 11	i	ii	iii
I	42,30	71,22	82,71
II	46,94	95,67	41,84
III	72,13	77,57	69,24

Game 12	i	ii	iii
I	35,57	87,40	22,24
II	75,49	50,70	46,36
III	16,75	17,43	61,82

Game 13	i	ii	iii
I	25,70	85,42	72,83
II	66,78	58,96	23,72
III	14,73	95,47	31,43

Game 14	i	ii	iii
I	42,89	45,19	72,52
II	59,37	77,18	51,77
III	26,24	84,63	38,48

Game 15	i	ii	iii
I	51,43	27,11	60,47
II	73,32	63,52	19,63
III	44,79	92,31	37,68

Game 16	i	ii	iii
I	71,40	66,22	50,63
II	34,95	55,66	14,30
III	82,47	35,76	46,54

Game 17	i	ii	iii
I	39,64	43,27	87,48
II	66,62	56,67	10,35
III	15,69	61,80	52,62

Game 18	i	ii	iii
I	31,47	84,65	51,60
II	68,43	73,19	48,70
III	52,91	66,56	39,14

Game 19	i	ii	iii
I	83,49	12,24	64,56
II	13,53	42,36	82,58
III	42,83	96,71	59,32

Game 20	i	ii	iii
I	32,59	58,82	56,64
II	71,96	36,42	24,12
III	83,42	53,13	49,83

Game 21	i	ii	iii
I	70,52	27,16	56,48
II	41,62	96,35	50,81
III	21,75	71,57	74,40

Game 22	i	ii	iii
I	54,72	77,23	64,43
II	18,29	59,73	37,98
III	50,58	42,76	83,52

Supplementary Figure 2. We report the list of games used in Assessment and Observation phases and the new games provided in the Re-assessment phase. For each game, we indicate the Nash equilibrium (cell with underlined payoffs), the column selected by the artificial agent (in red) and the row representing the participants' best-response (in green).

Experimental instructions

The following is a translation of the original instructions (in Italian). The experimenter read the instructions aloud to the participants while they followed along on their own copy.
We report in full the instructions of the Pre-feedback treatment. At the end of the section, we will highlight the differences in the instructions of the other two experimental treatments (No-feedback and Post-feedback). In the instructions, we refer to the three experimental phases (Assessment, Observation, Re-assessment) as Phase 1, Phase 2 and Phase 3.

Instructions (Pre-feedback treatment)

Dear participant

You are taking part in an experiment on interactive decisions. The decisions you make will determine a monetary reimbursement that you will receive privately at the end of the experiment, via bank transfer. The amount of money you will earn will depend on your decisions and those made by a computer that will choose based on a specific decision rule. This decision rule corresponds to the one most frequently used by people in strategic interaction contexts such as the one in which you are about to play. If you read these instructions carefully, you will have the opportunity to earn a considerable amount of money. The maximum possible payout can be € 20.00, the minimum € 9.00. No other participant will be informed of your payout. The experiment will consist of 3 phases. Each of the 3 phases of the experiment will be described to you in detail.

General information about the games

Each game phase consists of 22 rounds of interactive choice. In each round, you will be asked to choose one of 3 options: the term interactive means that the outcome of the decision you will make will depend both on your choice and the choice of the counterpart, which for all the duration of the experiment will consist of a Computer. The Computer will simulate the choices of a real player. This player was selected because their choice behavior corresponds to that most commonly used by a sample of about 300 participants who have previously played these same interactive games. In other words, in each of these games you are interacting with this prototypical player. In summary, the choices of the Computer will follow a very precise decision-making rule which is the one most commonly used by people in these interactive games. Computer choices will be the same for all participants in this experiment.

The structure of each game will be represented through a matrix like the one shown below:

Computer

Tu

	i	ii	iii
I	59<br>46	64<br>74	36<br>97
II	92<br>31	85<br>82	68<br>22
III	24<br>17	80<br>68	46<br>43

The game consists of 3 rows (I, II, III) and 3 columns (i, ii, iii). You will always play the role of ROW PLAYER, while the Computer will always play as the COLUMN PLAYER.

The possible choices of the row player (therefore, for you) are represented by the ROWS of the table (the first row at the top "I", the second row in the middle "II", the third row at the bottom "III"), while the choices of the column player (i.e., the Computer) are represented by the columns of the same table (the first column on the left "i", the second column in the middle "ii", the third column on the right "iii").

A cell within the matrix results from every possible combination of your choices and the Computer (i.e., for every possible combination of rows and columns in the table). Each cell shows two numerical values (one in green and one in orange). These values indicate the score attributed to each player (yours and that of the Computer) if the players have chosen that combination of actions. By convention, in each cell the bottom number colored in green always represents your gain, while the top number colored in orange always represents the gain of the Computer.

For example, referring to the matrix below, if YOU choose the first row at the top and the Computer the column in the middle, your respective scores can be found in the cell at the intersection of the selected row and column. In this example, the score is 74 points for you and 64 for the Computer, corresponding to 74 * 0.0035 = 0.259 Euros for you and 64 * 0.0035 = 0.224 Euros for the Computer. This means that for this game your winnings will be 0.259 Euros and that of the computer will be 0.224 Euros.

	i	ii	iii
I	59<br>46	64<br>74	36<br>97
II	92<br>31	85<br>82	68<br>22
III	24<br>17	80<br>68	46<br>43

Remember that you cannot directly choose the cell of the table, but only one of the rows, whereas the Computer will choose one of the columns. Only the combination of both choices will determine one and only one cell corresponding to yours and Computer's scores. Neither you nor the Computer will know in advance the choice of the counterpart. When you have made your decision, you will move on to the next game without receiving any feedback regarding the choice of Computer and the relative payoffs. Only at the end of the experiment, you will be able to know the total amount of the monetary reimbursement.
However, you know that you will always play with a Computer whose choices will follow a very precise decision rule which is the one most frequently used by players in these interactive games.

Phase 1: In Phase 1 you will be presented with 22 matrices, corresponding to 22 different interactive decisions. There is no relation among your choices in the different games: each game is independent from the others. To make your decision in a game, just click on the icons row "I", row "II" or row "III" and your selection will be marked in red. However, you will have the possibility to change your selection as many times as you want by simply clicking on a new row (I, II or III). To confirm your selection, you will be asked to press the "confirm" button located at the bottom of the screen.

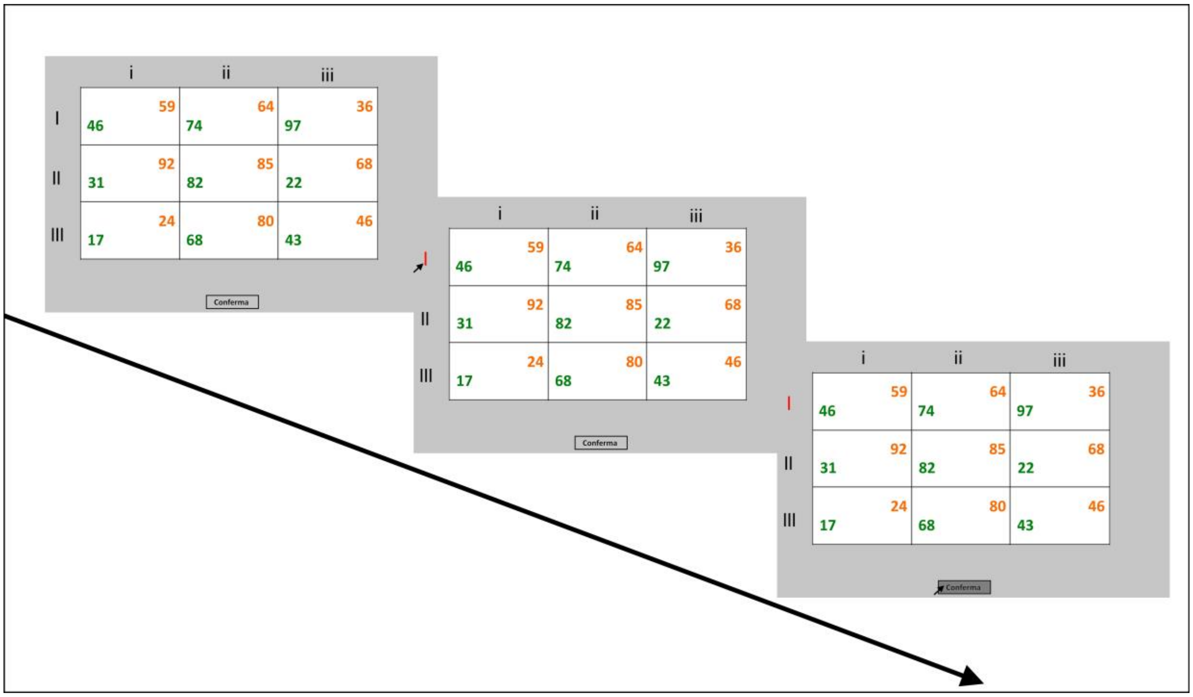

Once you have made your decision in each of the 22 games, Phase 1 will be over and you will be given the following information: you will be notified on your computer screen if your Phase 1 win corresponds to the highest score obtained in this experimental session or if another of the participants in this session got a bigger score. Once you have received this message you can proceed to Step 2.

Message presented at the end of Phase 1

Message A	Message B
Your score in Phase 1 corresponds to the highest score obtained in this experimental session	Your score in Phase 1 is lower than the highest score obtained in this experimental session

Phase 2: In Phase 2 you will be presented with the same 22 matrices that you played in Phase 1 and once again you will have to make your own decisions interacting with the Computer. The decision rule used by the Computer in Phase 2 will be the same as that used in Phase 1, so its choices in the 22 games will be the same as in the previous phase. However, pay attention that the order in which the 22 games are presented in each phase is random, so there will be no correspondence between the order of presentation in Phase 1 and that in Phase 2.

At this stage, you will receive an additional information. In each game, you will be able to observe the choice made in Phase 1 by the player who, in that phase, obtained the largest overall score. This information will appear on the right side of the screen in the form of an arrow. Once you have made your decision in each of the 22 games Phase 2 will be over and you will go straight to Phase 3 without receiving any additional information. So without knowing if your score in Phase 2 corresponds to the highest score obtained in this session or if another of the people present has obtained a higher score.

Phase 2: You will play the 22 matrices of Phase 1 again and in each game you will be able to observe the choice of the player who obtained the highest overall score in Phase 1. In this example, the arrow indicates that this player has selected row II.

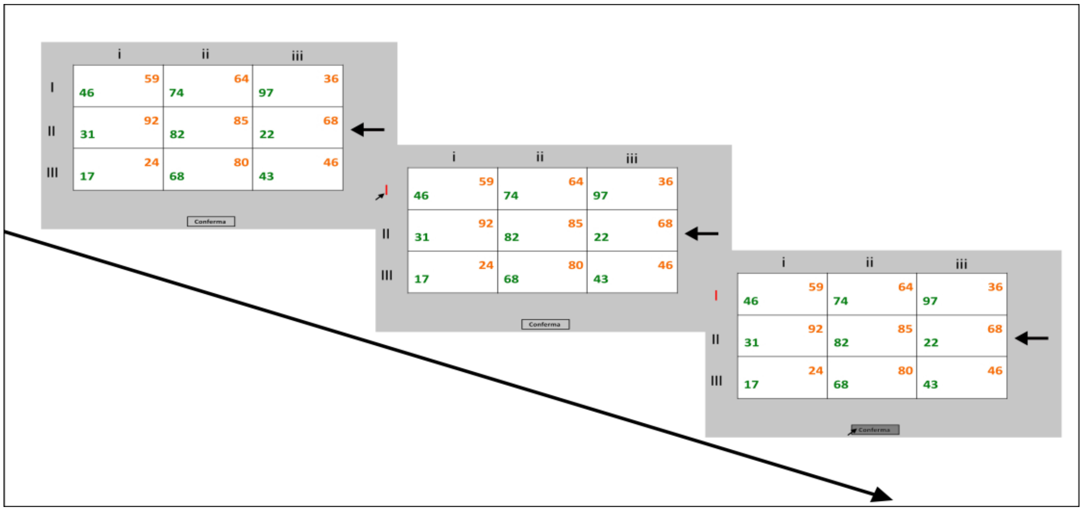

Phase 3: In Phase 3 you will be presented with 22 new matrices and once again you will have to make your own decisions by interacting with a Computer that will choose based on the decision rule most frequently used by people in this game context. You will not be given any additional information during this session. Once you have made your decision in each of the 22 games, Phase 3 will be over and you will be notified of your overall score and payout.

Phase 3: You will play the 22 matrices of Phases 1 and 2 again

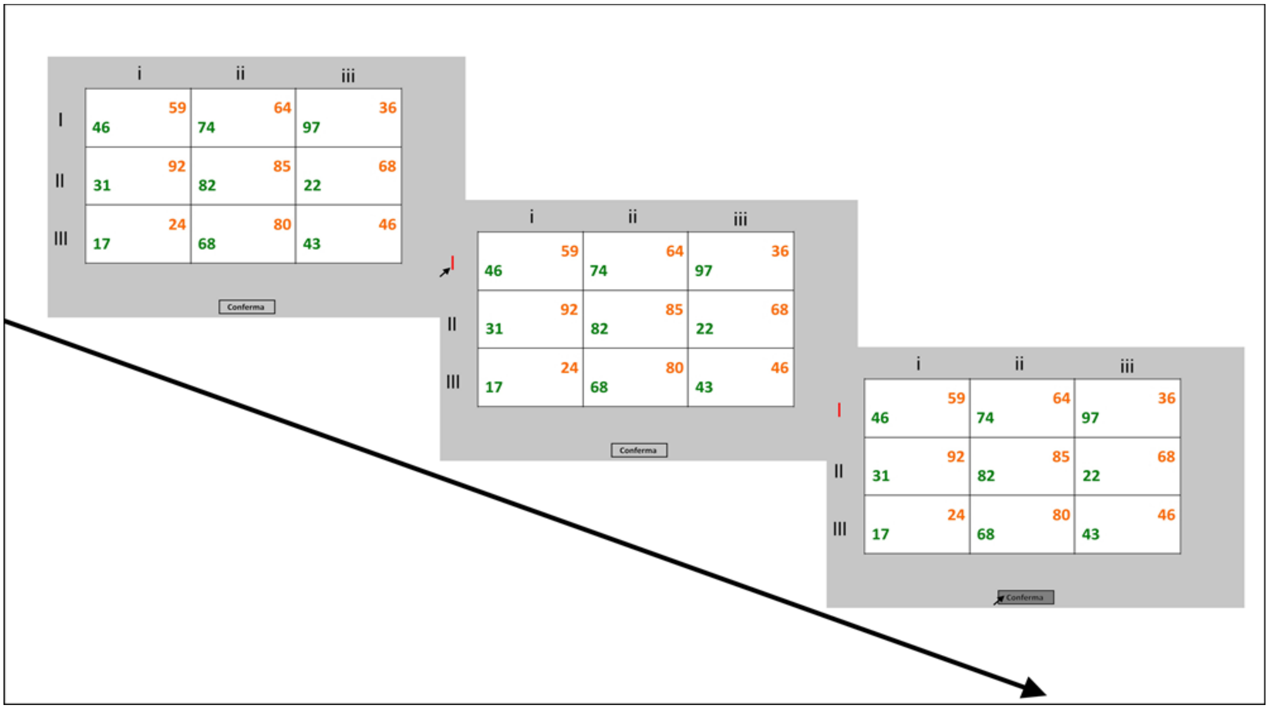

Score and Payout

Your winnings will be calculated as follows: for each of the 3 phases, the winnings obtained in the 22 games will be added together (3 sessions x 22 games = 66 games in total) and 3 Euros of participation will be added to the total.

Comprehension test and trial games

Before the experiment, you will be asked to answer a simple questionnaire to verify that the instructions are fully understood or if further clarifications are needed. At the end of the questionnaire, you will be asked to do a couple of trial games. However, these trial games are useful to familiarize with the graphical interface of the experiment. In these trial games, the computer will choose randomly and the winnings you will see on the screen at the end of the trial will not be credited to you.

If you have any doubts, feel free to raise your hand and ask for clarification.

Differences in the instructions for No-feedback and Post-feedback treatments.

<u>No-feedback:</u> Instructions were identical to those of the Pre-feedback treatment except for one important aspect. In Phase 2 (Observation phase), participants did not receive feedback on the choices of the best player in Phase 1 (Assessment phase), so this aspect was not present in the instructions.

<u>Post-feedback:</u> The only difference between Pre-feedback and Post-feedback treatments concerned the timing of the feedback on the choices of the best player in Phase 1 (Assessment phase). In the instructions of Phase 2 (Observation phase), this information was provided:

"In each game, after you have selected a row and confirmed your decision, you will be able to observe the choice made in Phase 1 by the player who, in that phase, obtained the highest overall score. However, you will not be able to change your choice following this new information. This information will appear on the right side of the screen in the form of an arrow. To proceed to the next game, just press the continue button, located at the bottom right of the screen."

Comprehension test

The following is a translation of the original comprehension test (in Italian) administered to participants after the experimental instructions. Two researchers checked the participants' test answers before starting the experiment and provided additional explanations in the case of mistakes or doubts.

Comprehension test (Pre-feedback & Post-feedback treatments)

Dear participant,

The following questionnaire serves only to verify that you understand the structure of the experiment and the choice task. We therefore ask you to answer the questions below.

If it helps you, you can consult the instruction sheet.

Thanks for your cooperation.

	i	ii	iii
I	5<br>6	6<br>4	3<br>7
II	9<br>1	8<br>2	6<br>2
III	2<br>7	8<br>8	4<br>3

Your role in the task will always be player: Row______ Column______

The role of the Computer in the task will always be player: Row______ Column______

In each round of play, you will choose: a Row____ a Column____ a Cell____

If in the matrix illustrated above you choose the third row and the Computer chooses the third column:

How many points do you collect? _______

How many points does the Computer collect? ________

What decision rule will be used by the Computer?

a) It will choose at random

b) It will choose the strategy least used by people in strategic interaction contexts

c) It will always choose the first Column

d) It will choose the strategy most used by people in strategic interaction contexts

How many phases does the experiment consist of?

One phase: Yes____No____

Two phases: Yes____No____

Three phases: Yes____No____

Four phases: Yes____No____

Once you have made your decision in a game.

1) Will you be able to see the outcome of the game? Yes__ No___

2) Will you be able to see your winnings? Yes__ No___

3) Will you be able to see the winnings on the Computer? Yes__ No___

What will you be told at the end of Phase 1?

1) If your score is equal to or less than the highest score obtained in Phase 1: Yes____No____

2) The overall score you collected in Phase 1: Yes____No____

3) The choices made by the Computer in Phase 1: Yes____No____

What additional information will you receive in each game during Phase 2?

1) You will be able to observe the choice of Computer: Yes____No____

2) You will see the choice of the Row player who in Phase 1 obtained the highest score: Yes____No____

3) You will be able to observe the choices you made in Phase 1: Yes____No____

When will you receive the information regarding the choice of the Row player who in Phase 1 obtained the highest overall score?

1) At each game round, before making your choice. Yes____No____

2) At each game round, after confirming your choice. Yes____No____

3) At the end of Phase 2. Yes____No____

What will you be told at the end of Phase 2?

1) If your score is equal to or less than the highest score obtained in Phase 2: Yes____No____

2) That Phase 2 is over and you will move on to Phase 3: Yes____No____

3) The overall score you collected in Phase 2: Yes____No____

What will you be told at the end of Phase 3?

1) If your score is equal to or less than the highest score obtained in Phase 3: Yes____No____

2) The overall score you collected in Phase 2: Yes____No____

3) Your overall score and payout including the winnings obtained in the three Phases plus the 3 Euros of participation: Yes____No____
